# The Trial of Intraoperative Cell Salvage Versus Transfusion in Ovarian Cancer (TIC TOC): Results of a Randomized Controlled Feasibility Study

**DOI:** 10.3390/cancers18040711

**Published:** 2026-02-22

**Authors:** Khadra Galaal, Patricia Jane Vickery, Elsa Marques, Joanne Palmer, Benjamin Jones, Emma O’Shaughnessy, Alberto Lopes, Paul Ewings, Ruud L. M. Bekkers, The TIC TOC Trial Group

**Affiliations:** 1 Gynaecology, Sultan Qaboos Comprehensive Care Cancer & Research Centre, University Medical City, Al Khoud, Muscat 123, Oman; 2 Peninsula Clinical Trials Unit, University of Plymouth, Plymouth PL4 8AA, UK; 3 Bristol Medical School, University of Bristol, Bristol BS8 1TH, UK; 4 NIHR ARC Southwest Peninsula (PenARC), University of Exeter, Exeter EX4 4QJ, UK; 5 Independent Researcher, Exeter EX4 4QJ, UK; 6 NIHR Research Design Service (South West), Exeter EX4 4QJ, UK; 7 Radboud University Medical Centre, 6525 GA Nijmegen, The Netherlands

**Keywords:** intraoperative cell salvage, blood transfusion, cytoreductive surgery, ovarian cancer, randomized feasibility trial

## Abstract

**Objectives:** To evaluate the acceptability and feasibility of intraoperative cell salvage (ICS) in women with ovarian cancer. A prospective multicenter randomized controlled feasibility trial. **Setting:** Four U.K. cancer centers. Women FIGO stage III/IV ovarian cancer supported by CT scan evidence. We randomized women to receive ICS or donor blood (as required) during surgery for ovarian cancer. The acceptability and feasibility of ICS in women with ovarian cancer having cytoreductive surgery; rates of recruitment for a larger trial and the likely completeness of resource use and outcome data; and blinding of allocation for participants and outcome assessors. A total of 57 women were included; the rate of recruitment was 1.4 cases per month, which closely aligns with the target, and 66% of the eligible patients were recruited. Overall, 91% of women completed the 30-day follow-up, and 75% completed the six-week follow-up. Mean (SD) blood loss in the ICS group was 1022 mL (SD 929 mL) and 924 mL (SD 646 mL) in the control group. A total of 16 (62%) of the participants undergoing surgery in the ICS arm received ICS reinfusion. Of the ten participants in the intervention group who did not receive ICS, six participants lost a significant volume of blood requiring transfusion. In the donor blood group, 14 of the 29 participants received donor blood. In the ICS group, 20/24 (83%) of participants, and 23/24 (96%) of research nurses did not know their group allocation. In the control group, 24/28 (86%) and 25/29 (86%) of participants and research nurses did not know the group allocation. Our study provides clinical evidence of the feasibility and acceptability of using ICS in ovarian cancer surgery and provides useful insights into the use of ICS within clinical trials. Women are open to having ICS as an alternative to blood transfusion. An appropriately powered randomized controlled trial is now required.

## Simple Summary

This study looked at whether it is acceptable to use intraoperative cell salvage (ICS) during surgery for women with advanced (stage 3–4) ovarian cancer. ICS is a method that collects a patient’s own blood lost during surgery, cleans it, and gives it back to them, reducing the need for donated blood. A total of 57 women with ovarian cancer took part; the amount of blood loss during surgery was similar in both groups. In the ICS group, about two-thirds of the women who had surgery received their own salvaged blood. Women appeared comfortable with the idea of receiving their own salvaged blood. Overall, this study shows that using ICS in ovarian cancer surgery is both feasible and acceptable to patients. The findings suggest that a larger trial should now be carried out to determine whether ICS can reduce the need for donor blood and improve patient outcomes.

## 1. Background

Ovarian cancer (OC) accounts for an estimated 295,414 new cases and 184,799 deaths worldwide annually [1]. Over 7600 women are diagnosed with OC in the UK each year [2,3,4]. Most women with OC are diagnosed when the cancer is already at an advanced stage [3,5]. Complete resection of all macroscopic disease has been shown to be the single most important prognostic factor in advanced OC [6,7,8]. Surgery is often extensive, and a complete resection of the tumor may require the removal of other solid organs [7,9]. Therefore, surgery for ovarian cancer can be associated with substantial intraoperative blood loss, and about 53% of patients lose more than 1.5 L during surgery [10,11]. Blood lost during surgery is conventionally replaced using donor blood transfusion, with the incidence of transfusion ranging from 35% to 77% [12,13]. Whilst often necessary and lifesaving, donor blood transfusion is associated with increased risks of a range of complications and adverse surgical outcomes, including wound, pulmonary, and renal complications, systemic sepsis, prolonged hospital stay, and death [14,15,16]. These observations of associated complications have contributed to the suggestion of a general transient depression of the immune system following transfusion of blood products, transfusion-induced immunomodulation (TRIM) [17]. In addition, higher rates of cancer recurrence were reported in those receiving transfusions during colorectal [18] and urological [19,20,21] surgery.

In advanced ovarian cancer, transfusion has been associated with a shorter time to recurrence and shorter survival [12,13,22]. An alternative strategy to donor blood transfusion is intraoperative cell salvage (ICS), which is the practice of recovering red cells from blood lost during surgery and returning them to the patient, thereby reducing or avoiding the use of donor blood [23]. As a blood conservation strategy, ICS is used where major blood loss is anticipated and has been used successfully in a wide range of surgical specialties, including urology, obstetrics, cardiothoracic, vascular, orthopedic, and hepatobiliary procedures [24,25,26]. Despite the lack of evidence of additional risk, ICS has not been widely used in cancer surgery because of the theoretical possibility of reintroducing malignant tumor cells into the patient’s bloodstream [27,28]. However, the use of leucocyte depletion filters in ICS is highly efficient at removing malignant cells [29]. Circulating tumor cells with the potential to form metastatic lesions are rare during ICS use [29,30,31]. Intraoperative ICS use has been shown to be associated with improved surgical outcomes in cervical and esophageal cancers [32,33,34]. However, few studies in ICS have been conducted, making it difficult for patients, clinicians, and health care organizations to make decisions about this technology.

The primary objective of this multicenter feasibility randomized controlled trial (RCT) was to evaluate the acceptability and feasibility of ICS in women with ovarian cancer having cytoreductive surgery. Secondary outcomes included estimation of (1) the likely rates of recruitment for a larger trial and the likely completeness of resource use and outcome data, (2) the ability to blind allocation for participants and outcome assessors, design data collection tools to collect resource use data, and confirmation of the resources required to run a larger definitive trial.

## 2. Methods

### 2.1. Study Design and Patient Recruitment

This multicenter, parallel-group feasibility RCT was conducted in four UK centers between February 2017 and August 2019; the trial took 30 months to complete. A detailed description of the trial methodology has been published elsewhere [35]. Women were eligible for the trial if a CT scan showed evidence supporting International Federation of Gynecology and Obstetrics (FIGO) stage III/IV ovarian or primary peritoneal cancer at presentation, with an Eastern Cooperative Oncology Group (ECOG) status of 0–1 [36,37]. Women who were pregnant or unwilling to accept donor blood or who had other concurrent malignancies or haemoglobinopathies were excluded from the study. The study conformed to the Declaration of Helsinki, and all participants provided written informed consent pre-operatively. We obtained ethics approval for the trial from the Southwest–Exeter Research Ethics Committee (ref.: 16/SW/0256).

### 2.2. Randomization and Masking

Women in each hospital were randomized after providing written informed consent; randomization was achieved by means of a web-based system built and maintained by the Peninsula Clinical Trials Unit using random numbers. Women were allocated to receive ICS reinfusion in the theater or donor blood transfusion using a 1:1 ratio with stratification by study site.

### 2.3. Intervention and Control

Blood lost in the operative field was collected, processed, and reinfused via a leucodepletion filter by the end of the surgical debulking procedure, either in theater or recovery. Participating sites were permitted to use any NHS-approved cell salvage machine, and salvaged blood samples were processed as described in the UK Cell Salvage Action Group’s quality control document [38] with study-specific ICS training provided prior to the study start.

Women allocated to the donor transfusion (control) group were considered for intraoperative transfusion in accordance with clinical judgment and guided by local hospital policy. The factors triggering transfusion (e.g., excessive blood loss, hypotension, reduced Hb) were recorded. In both treatment groups, women were able to receive donor blood intra-operatively or at any time after surgery to replace blood lost during surgery, as clinically indicated and in accordance with usual care [35].

### 2.4. Outcomes

Outcomes were centered around the feasibility of conducting a definitive RCT, both to inform the design of a future trial and to confirm the resources required to run it. This included assessing rates of participant recruitment and retention, completeness of outcome and resource use data, practicalities of randomization being conducted as late as possible, and the success of participant and assessor blinding. In addition, proposed outcomes for a definitive trial were collected, including health-related generic and cancer-specific quality of life measures [EuroQol 5 dimensions (EQ-5D-5L)], European Organization for Research and Treatment of Cancer core quality of life questionnaire [EORTC QLQ-C30] version 3, and EORTC quality of life questionnaire, ovarian cancer module [EORTC QLQ-OV28]. Clinical outcomes included the amount of donor blood given (total and ≤24 h post-surgery); visceral injury leading to excess bleeding; return to theater within 48 h; surgical site infection or thromboembolic complications within 30 days; number and nature of any other adverse events; and length of hospital stay and resource use. Safety data were reported as adverse and serious adverse events, and participants who discontinued the study were recorded, in addition to the reason for discontinuation.

### 2.5. Data Collection

Baseline data were collected before randomization and surgery; these included demographics, medical history, neo-adjuvant chemotherapy status, relevant current medications (specifically iron therapy or medications likely to affect bleeding), routine pre-operative blood results, quality of life, and resource use. Peri-operative data collection included operation details, blood loss, amount and nature of any transfusion/reinfusion, post-operative blood results, hospital length of stay, and any operative or post-operative complications. All women were followed up by telephone at 30 days to assess any post-surgical complications and by post at six weeks post-operatively to capture quality-of-life data and to record any further adverse events. Participants recruited early in the study were followed up at subsequent three-month intervals to a maximum of 10.5 months post-operatively.

### 2.6. Participant and Staff Interviews

Twelve women representing both treatment groups were interviewed by a researcher to elicit their views on the study, including acceptability of randomization, interventions, and follow-up. Selected participants were interviewed face-to-face approximately six weeks post-operatively and again, by telephone, approximately three months later. Surgeons participating in the study were invited to take part in a semi-structured interview, except for those at Leicester, as this site joined the study after completion of the qualitative element. Following informed consent, surgeons were interviewed by telephone by a member of the research team to explore their perceptions of the study, including the acceptability of randomizing patients to the trial interventions.

Safety data were reported as adverse and serious adverse events, and participants who discontinued from the study were recorded. Two investigators (KG and GH), blinded to treatment group allocation, made the assessment of cause of death, and classified it as treatment-related, disease-related, treatment- and disease-related, or other (non-ovarian cancer-, non-treatment-related).

### 2.7. Statistical Analysis

A Statistical Analysis Plan (SAP) was prepared in advance of database lock and approved by the research team, the Chief Investigator, and an independent Trial Steering Committee statistician. Statistical analysis was undertaken using Stata SE^®^ version 14.2 [39]. We aimed to recruit 60 patients for this feasibility study, which would provide robust information on likely recruitment and retention rates and enable full testing of the procedures involved in all trial processes. Specifically, 60 participants would allow estimation of binary outcomes such as retention rates with precision of approximately +/− 12 percentage points. Analyses were undertaken based on a modified Intention-to-Treat (ITT) principle, with participants analyzed as randomized without the use of an imputation method. As a feasibility study, TIC TOC was not suitably powered to be able to support conclusions drawn from any means of formal statistical analysis or hypothesis testing. As such, the statistical analysis is descriptive in nature with the intention of addressing the feasibility objectives and informing the design and conduct of a potential definitive trial.

**Figure 1:**
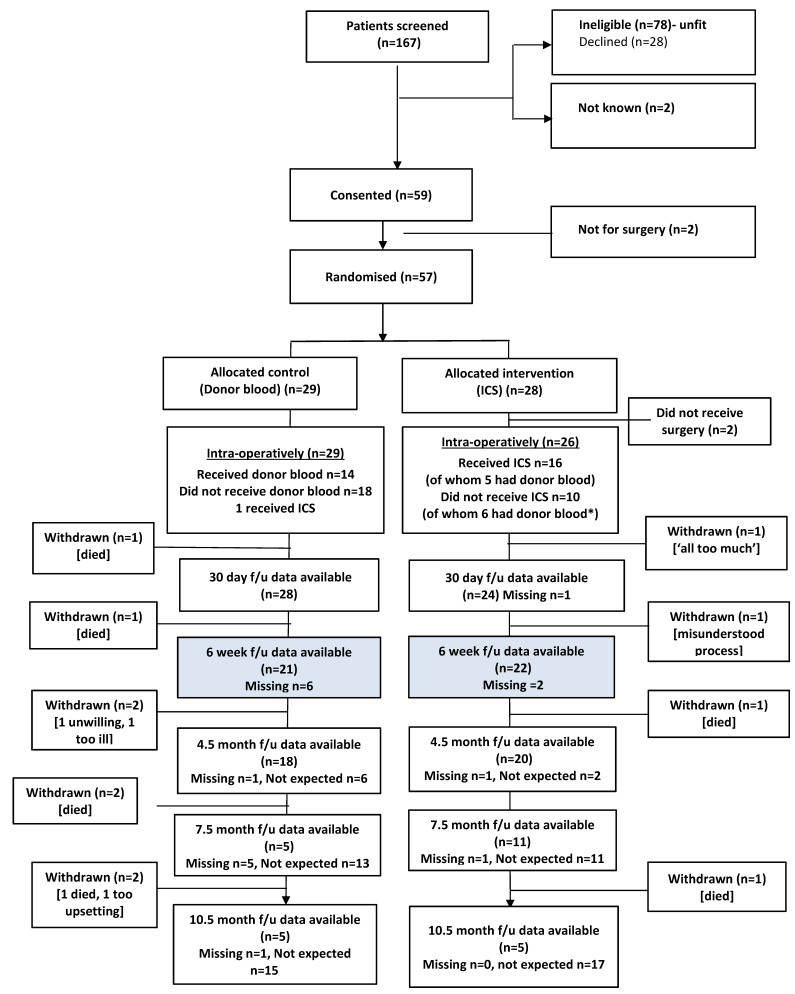
CONSORT diagram.

A CONSORT diagram (Figure 1) illustrates the flow of participants through the study, including numbers screened, declined/ineligible, consented, randomized, and followed up at each time point. Baseline data are summarized by allocated group and overall to informally check for balance between groups and therefore assess the success of the randomization procedure. Outcome measures are summarized by allocated group and overall as means and standard deviations for continuous outcomes and as counts and percentages for binary/ordinal data, and presented alongside 95% confidence intervals (CIs) where appropriate. Completeness of outcome and resource use data, and blinding success pertaining to both participants and outcome assessors, are presented as counts and percentages alongside 95% CIs by allocated group and overall.

In accordance with the journal’s guidelines, we will provide our data for the reproducibility of this study in other centers if such is requested.

### 2.8. Role of the Funding Source

The study funder had no role in study design, data collection, data analysis, data interpretation, or writing of this manuscript.

## 3. Results

### Participant Recruitment and Flow

The flow of participants through the trial is illustrated in the CONSORT diagram (Figure 1). A total of 167 women were approached between February 2017 and February 2018, of which 78 were ineligible and 59 provided written consent to participate. Two women were deemed not eligible for surgery following consent, and twenty-one declined participation in the study due to a lack of interest, being too busy, or being distracted by other events at the study time. Five of those approached were unwilling to undergo randomization, of whom three expressed a definite preference for cell salvage, and two had concerns about ICS and wanted to receive donor blood (Figure 1). Two participants in the ICS group did not proceed to full debulking surgery as planned because of the status of their disease, so they were withdrawn from the study soon after randomization and baseline assessment. Therefore, 57 women were randomized; however, 26 were allocated to receive ICS (two did not have surgery), and 29 were allocated to the control (donor blood).

**Figure 2:**
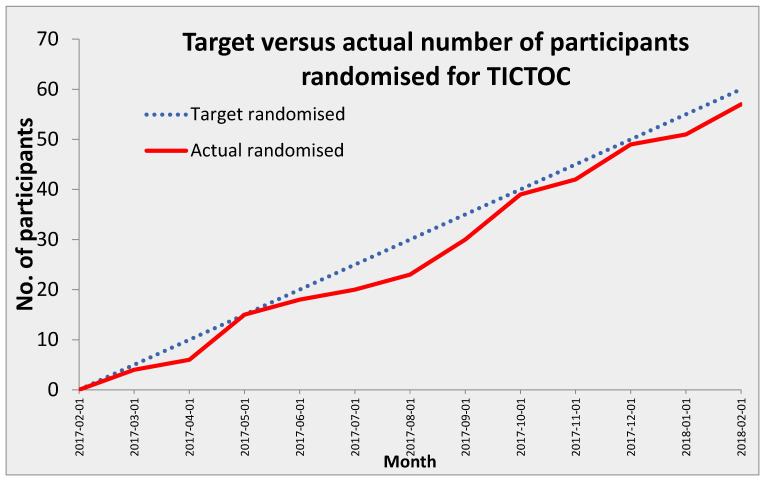
Actual recruitment.

**Figure 3:**
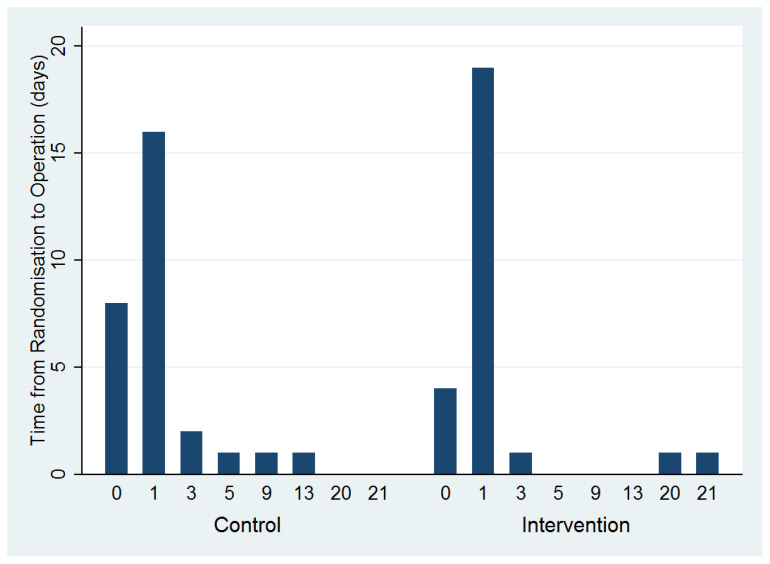
Time to randomization.

Recruitment rates varied across the four sites from 0.8 participants per month to 2.3 participants per month. The overall recruitment rate was 1.4 participants per site per month (Figure 2). The median time to randomization was one day for both groups; the range was 0–21 days for the ICS group and 0–13 days for the control group (Figure 3).

Baseline characteristics of randomized participants were generally well balanced between the groups (Table 1). The mean pre-operative hemoglobin level was 92 in the intervention group and 93 in the control group. After examination of the final histology, two participants in each trial arm were shown to have benign disease. It was recognized in advance that such occurrences may happen since it is not always possible to confirm a cancer diagnosis prior to surgery. For these participants, the cancer-related EORTC questionnaires were omitted from all subsequent follow-up points. An objective of this feasibility study was to explore the logistical practicality for theaters undertaking randomization as late as possible before surgery. The median interval between randomization and surgery was one day (range, 0–21 days). This lag between randomization and surgery was because two patients’ planned surgery was canceled and rescheduled after randomization.

Mean estimated blood loss in the ICS group was 1022 mL (SD 929 mL), and in the control group, it was 924 mL (SD 646 mL). Only 16 (62%) of the 26 participants undergoing surgery in the ICS arm received ICS reinfusion. Of the ten participants in the intervention group who did not receive ICS, four participants did not have enough blood collected intraoperatively into the ICS. However, six participants lost a significant volume of blood requiring blood transfusion both intraoperatively and within 24 h of surgery. The ICS line was dropped on the floor in one case, and no suitably trained staff were available in another. Furthermore, there was one reported problem with the ICS process in one case, which was rectified immediately during the operation (Table 2). However, in three cases, no information was provided as to why ICS was not used. Therefore, of the 26 ICS participants who had surgery in the ICS arm, 11 received donor blood, four intra-operatively only, three post-operatively only, and four both intra- and post-operatively.

In the donor blood group, 14 of the 29 participants received donor blood: five intra-operatively only, four post-operatively only, and five both intra- and post-operatively. The remaining 15 patients received no donor blood, although one was given ICS replacement following a clinical decision by the surgeon. Mean length of hospital stay in days, from operation until discharge, was 10.1 (SD 5.6) and 10.8 (SD 8.8) in the ICS and donor blood groups, respectively.

In the ICS group, 20/24 (83%) of participants, and 23/24 (96%) of research nurses did not know their group allocation. Similarly, in the control group, 24/28 (86%) and 25/29 (86%) of participants and research nurses did not know the group allocation.

Follow-up data were available for 52/57 (91%) participants (24 ICS, 28 donor) at 30 days post-operatively and for 43/57 (75%) participants (22 ICS, 21 donor) at six weeks post-operatively. As time allowed within the study, further data were available for 38, 16, and 10 participants at 4.5, 7.5, and 10.5 months post-operatively, respectively. Of the 55 participants who underwent surgery, seven subsequently withdrew from follow-up.

Reasons for withdrawal included misunderstanding of trial processes and follow-up, not feeling well enough to complete questionnaires, and feeling too tired. In addition, six died during the trial (Figure 1).

The donor blood administered during surgery was, on average, 2.8 units in the ICS arm and two units in the control arm. One participant in each group sustained an inadvertent visceral injury during surgery, and one participant in each group acquired severe infection (as defined in the study protocol) within 30 days post-operatively. No participants returned to the theater within 48 h.

Three participants (two ICS, one control) were diagnosed with a pulmonary embolism within 30 days post-operatively. Twenty-two (40%) participants (nine ICS, 13 control) experienced infections, mainly involving the surgical wound, drain site, abdomen, urinary tract, and chest. One patient in the control group developed a subphrenic abscess, and one admitted to the ITU with sepsis. One patient in the ICS group developed arrythmia post-operatively. Nine participants reported gastrointestinal disorders (five control and four ICS), including nausea, vomiting, diarrhea, constipation, ileus, and stoma problems. There were five episodes of pulmonary adverse events in eight (15%) participants, including pleural effusion (one), pulmonary embolism (three), and pneumothorax (one). Three patients experienced Pes; there was one ITU admission, one subphrenic abscess, one splenic hematoma, one wound infection with dehiscence, and one recurrent abdominal infection (Table 3). The six participant deaths (two ICS, four control) were all due to disease progression, including one complicated by pre-existing co-morbidities (Table 4).

## 4. Discussion

This feasibility RCT has demonstrated that women undergoing surgery for ovarian cancer were receptive to having ICS during surgery. This is the first trial assessing the acceptability of women and surgeons to using ICS for the replacement of blood lost during surgery for ovarian cancer. The rate of recruitment was 1.4 cases per month per site, which closely aligns with the expected targeted randomization (Figure 2), and 66% of eligible patients were recruited. In addition, 91% of women completed the 30-day follow-up, and 75% completed the six-week follow-up. Therefore, we have demonstrated that it is possible (and acceptable to patients and surgeons) to recruit and randomize women to both study arms, deliver trial treatments, maintain blinding, and collect appropriate outcome data. It is important to note that across multiple recent meta-analyses and new cohort data, intraoperative cell salvage (ICS) has not been shown to worsen recurrence or survival in cancer surgery [40], and in hepatectomy for hepatocellular carcinoma (HCC), it may even be associated with better oncologic outcomes [40] compared with allogeneic transfusion. However, almost all data are observational, and in most reports, washed blood + leukocyte-depletion filters (LDF) were used as well, and direct suction on the tumor was avoided [41]. In gynecological cancer, the evidence is scarce and suggests that ICS does not worsen oncologic outcomes. It is recommended that ICS be used with washed cells and LDF, ideally within a patient blood management framework [42].

This study provides clinical evidence of the safety of using ICS in ovarian cancer surgery with no increase in postoperative complications and provides useful insights into the use and evaluation of ICS within clinical trials. There was no difference in adverse events between the two arms of the trial.

With a significant number of ovarian cancer patients being of older age and presenting at a late stage, where management may be palliative [2,5], it is not surprising that the most common reason for ineligibility was lack of fitness for cytoreductive surgery. As a feasibility study, this trial was not powered to report on the impact of ICS on patients’ survival outcomes but to focus on the feasibility of conducting a definitive trial of ICS in ovarian cancer surgery and how such a trial might optimally be designed and delivered.

Several studies in a range of fields have demonstrated the ability of ICS to reduce the use of donor blood [42,43,44,45], but this is not universal; a recently published study of cesareans did not demonstrate any such reduction [46]. In our trial, we were unable to show a reduction in the use of donor blood transfusions. This may be because a good number of women had preoperative anemia (mean Hb 93), since some women had chemotherapy before surgery and had chemotherapy-related anemia. There was a high use of donor blood in both groups; we knew that it would happen for clinical reasons. However, in the ICS arm, we suspect that the surgical teams had increased reliance on using blood transfusions, which may be due to their lack of preparation in using innovations (ICS). It has been noted that without the appropriate amount of methodological expertise, it is difficult to transform the surgical culture into an evidence-seeking profession [47,48].

This feasibility study has highlighted some specific challenges for the prospects of running a definitive trial. One aspect for consideration is to improve the timing of randomization, to ensure that a participant is only randomized on the day of surgery [49]. Stringent training in the ICS protocol at study sites is also required to ensure that ICS equipment is not only available for those allocated to the intervention arm but is also used wherever possible.

A future trial would need enhanced training and monitoring to ensure the ICS use in the intervention group to its full potential. Even though all four sites had declared that they had previous experience with using ICS, it became clear that there were both logistical and training issues in the delivery of cell salvage during surgery. This was evident in cases where ICS was not used despite significant intraoperative blood loss, and perioperative donor transfusion was given instead. We therefore suggest that a technician with expertise in ICS equipment should be available in all cases.

The use of ICS is widespread across many surgical disciplines, where, at best, reduced use of donor blood has sometimes been demonstrated, but no definitive trial has conclusively demonstrated overall clinical benefit in cancer surgery [43,50,51,52]. A definitive trial is needed, but the choice of condition and surgery needs to be selected carefully: sufficient blood loss needs to be expected so that ICS can be used, but not so much loss that donor blood is also required. It may be possible to conduct a trial across different conditions, but the selection of outcomes would be challenging, and it is possible that ICS is beneficial in some areas and not in others.

## 5. Strengths and Limitations

- This feasibility randomized clinical trial is the first study evaluating the use of intraoperative cell salvage in cytoreductive surgery for ovarian cancer [35].
- A multicenter RCT design was utilized, incorporating robust protocol and oversight, as well as previously published methodology.
- Data on acceptability, recruitment, retention, and adherence advance the knowledge and literature on the use of ICS in ovarian cancer patients.
- The study recruited patients with advanced ovarian cancer deemed eligible by experienced clinicians based on clinical findings and investigation results. This approach can be subjective and lead to variability in ovarian cancer. However, this is in keeping with the pragmatic aim of our clinical trial.
- This trial had a small sample size and was not powered or designed to assess the effects of ICS in advanced ovarian cancer. The findings should therefore be considered preliminary.
- Only 62% of patients randomized to ICS received salvaged blood.
- There was heterogeneity in the transfusion policies between the different centers.

## 6. Conclusions

Our study showed that it is feasible and acceptable to use ICS in ovarian cancer surgery and provides useful insights into the use and evaluation of ICS within clinical trials.

Women with ovarian cancer were open to having ICS as an alternative to blood transfusion. ICS might work as an adjuvant to donor blood instead of an alternative. Barriers to a definitive trial include staff and ICS machinery availability in the theater. An appropriately powered randomized controlled trial is now required to address these barriers and investigate the oncological impact of intraoperative cell salvage in women with ovarian cancer.

## Figures and Tables

**Table 1:** Baseline characteristics and demographics of randomized participants.

Variable Mean (SD) [Range] Unless Otherwise Stated	Intervention (ICS) (*n* = 26)	Control (*n* = 29)
Age (years)	65.7 (10.8) [43, 80]	63.6 (11.0) [42, 82]
BMI (kg/m^2^)	27.2 (6.2) [19.0, 39.4]	27.1 (7.5) [18.1, 46.0]
Histology of epithelial ovarian cancer	25 (95%)	27 (93%)
Hemoglobin	93.4 (8.5) [76.2, 113.2]	92.3 (5.9) [77.9, 102.7]
WBC	9.0 (7.0) [3.2, 36.3]	7.0 (2.5) [3.7, 15.2]
Smoking, ***n*** (%)		
Current smoker	2 (7.7%)	3 (10.3%)
Ex-smoker	11 (42.3%)	6 (20.7%)
Never smoked	13 (50%)	20 (69.0%)
ECOG score, ***n*** (%)		
0	13 (50%)	14 (48.3%)
1	13 (50%)	15 (51.7%)

**Table 2:** Baseline variables and outcomes in the ICS group.

Baseline Variable Mean (SD) [Range] Unless Otherwise Stated	Intervention (ICS) (*n* = 26)
Surgical complexity score	*n* = 24 3.9 (2.6) [0, 9]
**Pre-op iron therapy, *n* (%)**	3 (11%)
Medication that may affect bleeding, ***n*** (%)	10 (38%)
Received pre-op blood transfusion, ***n* **(%)	2 (7.6%)
Received neoadjuvant chemotherapy, *n* (%)	11 (42.3%)
Preoperative blood transfusion	2 (7.69%)
Neoadjuvant chemotherapy, *n* (%)	11 (42.3%)
Blood loss mean (SD)	1022 mL (929)
Received ICS blood, *n* (%)	16 (61.5)
Received donor blood during surgery, *n* (%)	8 (30.7)
Units of donor blood during surgery, median (IQR)	2 (1.5, 4)
Length of hospital stay, median (IQR)	9 (6, 11)

**Table 3:** Summary of adverse events occurring in the study.

Complications (Adverse Events)	Control 29	Intervention (ICS) 26
Infections, *n* (%)	13 (44.8)	9 (34.6)
Severe infection, *n* (%)	2 (6.89)	1 (3.8)
Thromboembolic complications, PE *n* (%)	2 (6.89)	1 (3.8)
Visceral injury, *n* (%)	1 (3.44)	1 (3.8)
Cardiac disorders (arrhythmia), *n* (%)	0	1 (3.8)
Vascular disorders, *n* (%)	1 (3.44)	0
Respiratory, thoracic, and mediastinal disorders, *n* (%)	1 (3.44)	5 (19.2)
Gastrointestinal disorders (nausea/vomiting), *n* (%)	7 (24.1)	4 (15.3)
Skin and subcutaneous tissue disorders, *n* (%)	1 (3.44)	1 (3.8)
Renal and urinary disorders, *n* (%)	1 (3.44)	1 (3.8)
Other, *n* (%)	3 (10.3)	1 (3.8)

**Table 4:** Summary of serious adverse events occurring in the study.

Allocation	Description of Event	Cause of Death	PI Opinion of Relatedness	Second Opinion
Intervention	Death	Disease progression	Not related	Not related
Intervention	Death	Disease progression	Not related	Not related
Control	Death (poor post-op recovery complicated by Parkinson’s disease)	Respiratory arrest	Not related	Not related
Control	Death	Disease progression	Not related	Not related
Control	Death	Disease progression	N/A	N/A *
Control	Death	Disease progression	Unlikely	Unlikely

## Data Availability

Data are contained within the article.

## Appendix A

TIC TOC Trial Group: Khadra Galaal, Jane Vickery, Elsa Marques, Joanne Palme, Ben Jones Emma O’Shaughnessy, Alberto Lopes, Paul Ewing, Catherine Ralph, Colin Pritchard, Jennifer Wingham, John Faulds, Andy Barton, Michael Fisick, Geoffrey Hughes, Ahmed Talaat, Sophia Julian, Raj Naik, Peter Ricketts, Chris Thorpe, Steve Christian, Fiaz Ahmed, Ann Fisher, Christine Ang, Ali Kucukmetin, Nithya Ratnavelu, Lorraine Pierce, Wendy McCormick, Beverly McClelland, Supratik Chattopadhyay.
